# Dynamic of active microbial diversity in rhizosphere sediments of halophytes used for bioremediation of earthen shrimp ponds

**DOI:** 10.1186/s40793-023-00512-x

**Published:** 2023-07-12

**Authors:** Marie Colette, Linda Guentas, Luc Della Patrona, Dominique Ansquer, Nolwenn Callac

**Affiliations:** 1 French Institute for Research in the Science of the Sea (IFREMER), Research Institute for Development (IRD), University of New Caledonia, University of Reunion, CNRS, UMR 9220 ENTROPIE, Noumea, New Caledonia; 2 Institute of Exact and Applied Sciences (ISEA), EA 7484, University of New Caledonia, Noumea, 98851 New Caledonia

**Keywords:** Halophytes, Bioremediation, Shrimp farming, Sediment microbiota, Specific microbiota, Metabarcoding 16s RNA, Denitrification

## Abstract

**Background:**

In New-Caledonia, at the end of each shrimp production cycle, earthen ponds are drained and dried to enhance microbial decomposition of nutrient-rich waste trapped in the sediment during the rearing. However, excessive ponds drying may not be suitable for the decomposition activities of microorganisms. Halophytes, salt tolerant plants, naturally grow at vicinity of shrimp ponds; due to their specificity, we explored whether halophytes cultivation during the pond drying period may be suitable for pond bioremediation. In addition, plants are closely associated with microorganisms, which may play a significant role in organic matter decomposition and therefore in bioremediation. Thus, in this study we aimed to determine the impact of 3 halophyte species (*Suaeda australis, Sarcocornia quinqueflora* and *Atriplex jubata*) on active sediment microbial communities and their implications on organic matter degradation.

**Results:**

Drying significantly decreased the microbial diversity index compared to those of wet sediment or sediment with halophytes. Microbial profiles varied significantly over time and according to the experimental conditions (wet, dry sediment or sediment with halophyte species). Halophytes species seemed to promote putative microbial metabolism activities in the sediment. Taxa related to nitrogen removal, carbon mineralisation, sulphur reduction and sulphide oxidation were significant biomarkers in sediment harbouring halophytes and may be relevant for bioremediation. Whereas microbial communities of dry sediment were marked by soil limited-moisture taxa with no identification of microbial metabolic functions. Nitrogen reduction in sediments was evidenced in wet sediment and in sediments with halophytes cultures, along with putative microbial denitrification activities. The greatest nitrogen reduction was observed in halophytes culture.

**Conclusion:**

The efficiency of sediment bioremediation by halophytes appears to be the result of both rhizosphere microbial communities and plant nutrition. Their cultures during the pond drying period may be used as aquaculture diversification by being a sustainable system.

**Supplementary Information:**

The online version contains supplementary material available at 10.1186/s40793-023-00512-x.

## Introduction

In many shrimp farms, drainage and drying of shrimp earthen ponds at the end of each production cycle allow aeration of pond bottom sediment in order to enhance microbial decomposition of accumulated organic matter [1]. Indeed, in shrimp farming, as in many other aquaculture systems, feed pellets are daily distributed but are not totally assimilated by farmed species. They therefore tend to accumulate in pond bottom sediments, along with faeces and dead plankton [2–4]. Excessive organic matter accumulation at the pond bottom can exceed the microbial mineralization capacity of the system, resulting in anaerobic conditions and in the release of toxic metabolites (ammonia, nitrite and hydrogen sulphide) that could affect the health and survival of farmed species [2–4]. However, complete pond drying at the end of the rearing may not be suitable for microbial communities as water stress could lead to a decrease in bacterial decomposition activity [2, 5]. In addition pond drying consume time that could be used to produce shrimp along with economic profits [2].

Since the last decade, integrated aquaculture farming systems emerge as a more sustainable way to produce by reducing nutrient excess and organic matter generated from aquaculture activities [6, 7]. These systems rely on resources optimization (i.e.: space, water, feed and management), and the waste from one system may be used as input to another [6]. Aquaculture wastes typically contain ammonia, nitrate, and phosphorus, which are essential nutrients for plant growth. Incorporation of agriculture to aquaculture is an integrated system where aquaculture wastes are used to produce plant biomass thereby reducing the wastes concentrations in the farming system (e.g. water or sediment) [8–10]. However, plant tolerance to salt is a key limiting factor for their integration into marine or brackish aquaculture systems, as salt is the main stressor for plants. Marine and brackish aquaculture systems are therefore limited to the use of salt tolerant plants, known as halophytic [11]. Due to their salinity tolerance, the integration of halophytes in marine aquaculture systems with the aim of remediating nutrient-rich waste products, has received growing attention in the past few years [12–14]. The efficiency of halophyte bioremediation has been proven in aquaponics and sand-substrate systems, with significant removal of aquaculture wastes [14, 15]. Integrated shrimp-plant culture is a topic of recent interest and shrimp-vegetable rotational farming system with vegetable culture in tidal ponds during the idle period may be an efficient way to reduce nutrient accumulation in ponds sediments during shrimp rearing [11, 16, 17]. In the soil, most nutrients, such as N, P and S, are bound to organic molecules and are minimally bioavailable to plants. Thus, soil microbial transformation is crucial for plant nutrition as microorganisms possess metabolic pathways to break down organic matter and to convert it into inorganic forms available for plant nutrition [18, 19]. Thus, the significant influence of microorganisms on soil nutrient cycling should be taken into account in sediment nutrient removal efficiency from shrimp-vegetable rotational farming systems. However, little is known about the dynamics of microbial communities in sediment hosting shrimp-vegetable rotational farming systems [20]. The development of modern genomic tools has accelerated the study of microbial community structure which was earlier dependent on culture technologies; as indeed, less than 1% of the microorganisms are cultivable [21, 22].

In soil, microbial communities are significantly influenced by plant roots as they provide adhesion sites for microbial attachment and energy source for most heterotrophic microorganisms through the release of carbon compounds [18]. It was estimated that 10 to 44% of the photosynthetic carbon fixed by plants could be transferred to the rhizosphere. Hiltner defined the rhizosphere as the soil volume under the influence of roots [23]. Indeed, roots exudates [24], dead plant material and residues represent significant sources of energy for soil heterotrophs growth [18]. Thus, the rhizosphere is often reported as an hot spot of microbial activities [25–27]. Therefore, understanding how plants shape their surrounding microbial diversity may provide fundamental information on rhizosphere soil processes such as nutrient cycling.

In New Caledonia shrimp farming is principally semi-intensive and takes place in earthen-ponds excavated into bare saltpans upstream of mangrove forests [28]. Shrimp-farming has an important economic impact as it represents the second largest export sector of the island and contributes to economic development in remote areas [29]. However, since few years, shrimp farming face important production decrease, from a peak at 2500 t in 2004 to less than 1500 t nowadays leading to a negative economic impact (FAO data base “Fisheries and Aquaculture). This production drop is due to both seasonal vibriosis affecting adults reared in pond, and post-larvae deficits due to larvae mortalities in hatcheries [30, 31]. Poor pond bottom conditions (anaerobic conditions, sludge) are favourable factors to the occurrence of *Vibrio* species and to their development in the sediment. Thus, shrimp farmers are looking for solutions to restore the production capacity of the ponds (shrimp farmers, personal communication) [29]. In New Caledonia, most of the upper parts of the shrimp pond, and particularly the internal dikes, are colonised by halophyte species able to grow in environments with extreme salinity. Therefore these species may appear as a solution for bioremediation of accumulated organic matter in shrimp pond sediments [16].

In our study, we explored the bioremediation potential of halophyte culture and their microbiota in shrimp earthen pond sediments during the idle period. For that, we have conducted an experimental greenhouse study where three halophyte species with high economic potential: *Sarcocornia quinqueflora, Suaeda australis* and *Atriplex jubata*, were separately grown in pots filled with shrimp earthen pond sediment. As bioremediation may involve microorganisms of the earthen pond soil, we aim to answer (1) what are the differences in the removal efficiency of accumulated organic matters and nutrients between different treatments (dry, wet, and plant cultivation), and (2) whether these differences are related with the sediment microbes. For this last question, we explored the active microbial diversity present at a specific time in sediments colonized or not by halophyte species, through cDNA sequencing of the V4 region of the 16 S RNA gene. We first explored the alpha diversity indexes and evidenced significant lower values in dry sediment compare to wet sediment and sediment with halophytes. The hierarchical clustering dendrogram evidenced dissimilarities among the different microbiotas related to experimental conditions: wet, dry or with halophytes, and according to the experiment time. Then, we focused on the microbiotas of each experimental conditions and associated them with ecological soil functions.

## Materials and methods

### Greenhouse experiment

The study took place in an experimental greenhouse located at the Aigue Marine shrimp farm at Boulouparis, New Caledonia, bordered by the Saint Vincent Bay. The greenhouse was equipped with a shade house. The experiment extended from September (2021) the inter-season period, to February (2022) the hot season. Atmospheric parameters were recorded every hour by a weather station (HOBO 0664 H21-USB, ONSET®, Cape Cod, MA, USA) [32]. The mean daily temperature in September was 25 °C during the daytime (am) and 21 °C the night time (pm), the mean relative humidity was 68% the am to 82% the pm. In October to November the daily temperature was higher with 28 °C the am and 23 °C the pm, and the mean relative humidity was 60% the am and 77% the pm. In December to January the daily temperature was 29 °C the am and 25 °C the pm with relative humidity at 69% am and 84% the pm.

Sediments from the shrimp ponds were collected on the 18th August 2020 with a mid-size excavator at the end of the rearing period during the pond-drying period. The collected sediments were transported to the greenhouse and stored for a few days until they were poured into the 42 L pots. The seedlings were obtained from the germination of seeds from mother plants of three different halophyte species; *Sarcocornia quinqueflora* (SarQ), *Suaeda australis* (SuaA) and *Atriplex jubata* (AtrJ). Mothers’ plants were grown in another experimental greenhouse located in New-Caledonia. The seeds were cultivated in a greenhouse, in a mixture of sand and potting soil during two months. Then, the 2 months old seedlings were separately transplanted into the 42 L pots previously filled with shrimp pond sediment, and 9 pots per halophyte species were used for their cultivation. To ensure minimum survival, 3 to 4 young seedlings per pot were planted. In addition, young seedlings were transplanted with a part of their initial growth substrate to avoid drastic change. The old seedlings were grown for 6 months in a greenhouse and exposed were irrigated daily with an automatic watering. The seawater of the lagoon was pumped the day before each watering directly into the bay of Saint-Vincent, where the farm is located, and stored in a tank of about 500 L. Then, twice a week, all the halophytes were watered with the lagoon seawater. The same natural water is used for the shrimp farm and the plant watering. The control treatments consisted of pots with pond sediment only, maintained under dry or wet conditions (same watering conditions as for the plants). Each control treatment was made in triplicate using 3 pots of 42 L. The dry conditions consisted of placing the pots outside the greenhouse to remain dry alike the pond sediment during the drying period of the pond. During the drying period, the bottom of the emptied ponds is dried in the sun [28, 33]. By placing the pots outside the greenhouse, sediments were exposed to the same meteorological conditions (e.g.: raining event, hot, sun) than the emptied pond sediment.

### Sampling

All the equipment used for sediment sampling was cautiously cleaned with ethanol. The first 2–3 cm of sediment were collected aseptically using RNA/DNA free gloves and spatula and transferred into RNA/DNA free 50 ml tubes. In addition, in order to avoid sediment contamination due to the coring, care was taken to collect only the inside fraction of the sediment (meaning the fraction that was not in contact with the push-core).

For each modality, we have sampling sediment in triplicates from the same 42 L pots. In order to ensure sampling homogeneity, each sediment sample taken was made up of a pool of 3 to 4 samples per pot. Sampling was carried out during the 6 months of cultivation on day 0 (D0), day 30 (D30) and day 150 (D150) for all conditions (dry, wet, halophytes). We chose 6 months period for our experimentation as it is the duration of pond sediment drying practiced by shrimp farmers in New Caledonia. In New Caledonia, the shrimp production is seasonal and reaches its peak from March to June. This seasonality leads some farmers to realize only one production cycle in the year and to leave the pond empty for several months [34].

For the halophyte *Suaeda australis* solely, sediment samples were also collected after 60 days (D60) and 90 days (D90) of cultivation. After collection, samples were stored at 4 °C during transport to the laboratory and frozen at − 80 °C until further processing.

### RNA extractions, retro transcription and sequencing

For each sediment sample, RNA was extracted using RNA PowerSoil Total RNA Isolation Kit (MoBio Laboratories, Inc.) according to the manufacturer’s instructions. Total RNAs were first reverse-transcripted into complementary DNA (cDNAs) as described in Callac et al., (2022) [35]. Then, 20 µL of these first strand cDNAs were directly used to perform the second strand cDNA synthesis using the Second Strand cDNA Synthesis Kit (Invitrogen) following the manufacturer’s protocol. All cDNAs were sent to MrDNA (Shallowater, Texas, United States) where PCR using the 515f-806R primers [36], barcode indexing and sequencing of the V4 hypervariable region of the reverse-transcripted procaryotic 16 S ribosomal RNA molecule were conducted. We chose to amplify the V4 hypervariable region as recommended by the Earth Microbiome Project (https://earthmicrobiome.org/protocols-and-standards/16s) to detect both *Archaea* and *Bacteria* and because this region is widely used to study soil microbiomes [37]. In addition, the prokaryotic primer pair 515 F-806R was reported to provide a great depth and taxa coverage for frameworks emphasizing ecological relationships between soil, plant, animal and human health [37].

The sequencing was done with an average of 20 k raw reads per sample. The raw 16 S RNA data are available in the NCBI SRA repository under the BioProject ID PRJNA925577: HALOREMED (submission: SUB12696467, SRA accession numbers from SAMN32802582 to SAMN32802610).

### Data analysis and preparation

#### Amplicon analysis

The raw reads were processed using the DADA2 [38] package available in the Rstudio software, where all the sequences with a quality score above 30 were kept. The sequences were filtered and trimmed with the following parameters: a maximum excepted error (maxEE) at 2, a maximum N (maxN) at 0, a truncation based on quality scores (truncQ) at 2, trimLeft at 19 pb and 20 pb to remove the primers and truncLen set at 240 pb for the forward reads and 180 pb for the reverse reads to remove low quality tails. The chimeras were removed using the consensus method, and the taxonomy was assigned using the Silva 138 SSU Ref NR99 database [39]. Prior to further analysis, sequences with no affiliation or affiliated to the Eukaryota, Mitochondria or Chloroplasts were removed from the ASV table.

#### Alpha diversity

The alpha diversity was estimated using the richness indices of ACE (Abundance-based coverage estimator) and Observed ASV calculated on RStudio software with the **microeco** package. Definition of this different indices can be found in Hughert and Anderson 2017 [40]. The observed ASV represent the number of different ASVs in a sample. Whereas, the ACE index (Abundance-based Coverage Estimator) estimate the richness of a sample by taking account the number of singletons, doubleton and rare OTUs (generally less than 10 sequences) [40, 41]. We also used the Inverse Simpson and Exponential Shannon indices from Hill numbers (*q* = 1 and *q* = 2) with function *renyi* from **vegan package**. The Hill numbers was recommended to a reliable estimation of the microbial richness and diversity [42, 43]. Then a non-parametric test of Kruskal-Wallis followed by a Dunn test was performed with RStudio (**dunn.test package**) to show significant differences between experimental conditions (dry, wet, halophyte species*)* and sampling time (D0 and D150 only). Prior to downstream microbial analysis, data were then normalized with the Counts Per Million (CPM) method using the cpm function available in the edgeR package under RSudio, allowing to normalized all the libraries to 1.000.000 reads as described in Giraud et al., (2021;2022) and Callac et al., (2022;2023) [30, 35, 44]. We used the CPM normalization, method to normalize gene expression, as we have extracted RNA and then used cDNA to sequence the V4 region of the 16 S RNA gene to investigate the active microbiota of the sediments.

#### Beta diversity

The beta-diversity was investigated by building a dendrogram based on a Bray-Curtis dissimilarity matrix and Ward method [30, 35]. It was performed on MicrobiomeAnalyst to clustered sediment samples following dissimilarity in their microbial profiles [45]. Then, stacked bar plots of relative abundance of microbial communities were done to evidence the microbial profiles of the sediment samples. Stacked bar plot and relative abundance values were obtained on MicrobiomeAnalyst web tool.

#### Venn diagram

Following the clustering results from the dendrogram (see results section), only the core microbiota of each replicate collected at D150 were kept to construct a Venn diagram. Venn diagrams were made using the open-source component for web environment Jvenn (http://jvenn.toulouse.inra.fr). The specific microbiotas of dry, wet and halophytes (SarQ, SuaA, AtrJ) sediments were represented with stacked bar plots the family level.

#### LEfSe

A Linear discriminant analysis (LDA) Effect size (LEfSe) analysis [34] was performed to identify discriminative features at the genus levels using the specific sediment microbiota of the dry and wet sediment and sediment harboring halophytes species. LEfSe was performed with a threshold set at 3.75 using microeco R package on RStudio [47].

#### Sediment functional community profiling

Relevant biomarkers of sediment specific microbiomes were used to make functional annotation of 16 S ribosomal RNA bacterial gene sequences from the SILVA database. The *microeco* R package was used to assign putative ecological function of the previously evidenced taxa from the LEfSe. We used Tax4fun R package [48] to determine the metabolic pathway prediction of the microbial communities and FAPTROAX v1.2.4 database [49] to make a Functional Annotation of Prokaryotic Taxa. Tax4Fun predict microbial communities function as phenotypes of gene families or enzymes activities based on gene content, whereas FAPROTAX predict metabolic phenotypes and ecologically relevant functions based on the literature of cultured taxa [49, 50]. FAPROTAX was reported as a helpful tool to highlight functions related to biogeochemical dynamics especially on N and C cycles [50]. It was, for instance used in Ji et al. (2022), to predict bacterial functions in Korean pine root tips and rhizosphere soil [51]. Tax4fun is suitable to target changes in gene expressions or in potential enzymatic activities. Although these tools cannot replace the functional assessment *via* metagenomic or metatranscriptomic shotgun sequencing, as they provide insights into functional capabilities of prokaryotic communities in diverse habitats [52].

### Soil chemistry analysis

Total organic carbon of the sediment was determined using the Walkley and Black method [53] with hot sulphuric acid and potassium dichromate. Nitrate and ammoniums were extracted from the sediment with KCl 1 N solution. Nitrate concentration (NO_3_^−^) was evaluated by colorimetric method based on the Griess reaction and Ammonium concentration in the sediment was determined using the Nessler method (ISO 14256-2:2005). Sediment analyses were performed by the Laboratory of Analytics Means (LAMA/ISO 9001, Noumea, New Caledonia). A non-parametric test of Kruskal-Wallis followed by a Dunn test was performed with RStudio (dunn.test package) to show statistically significant differences between the experimental conditions.

## Result

### Alpha diversity

**Fig. 1:**
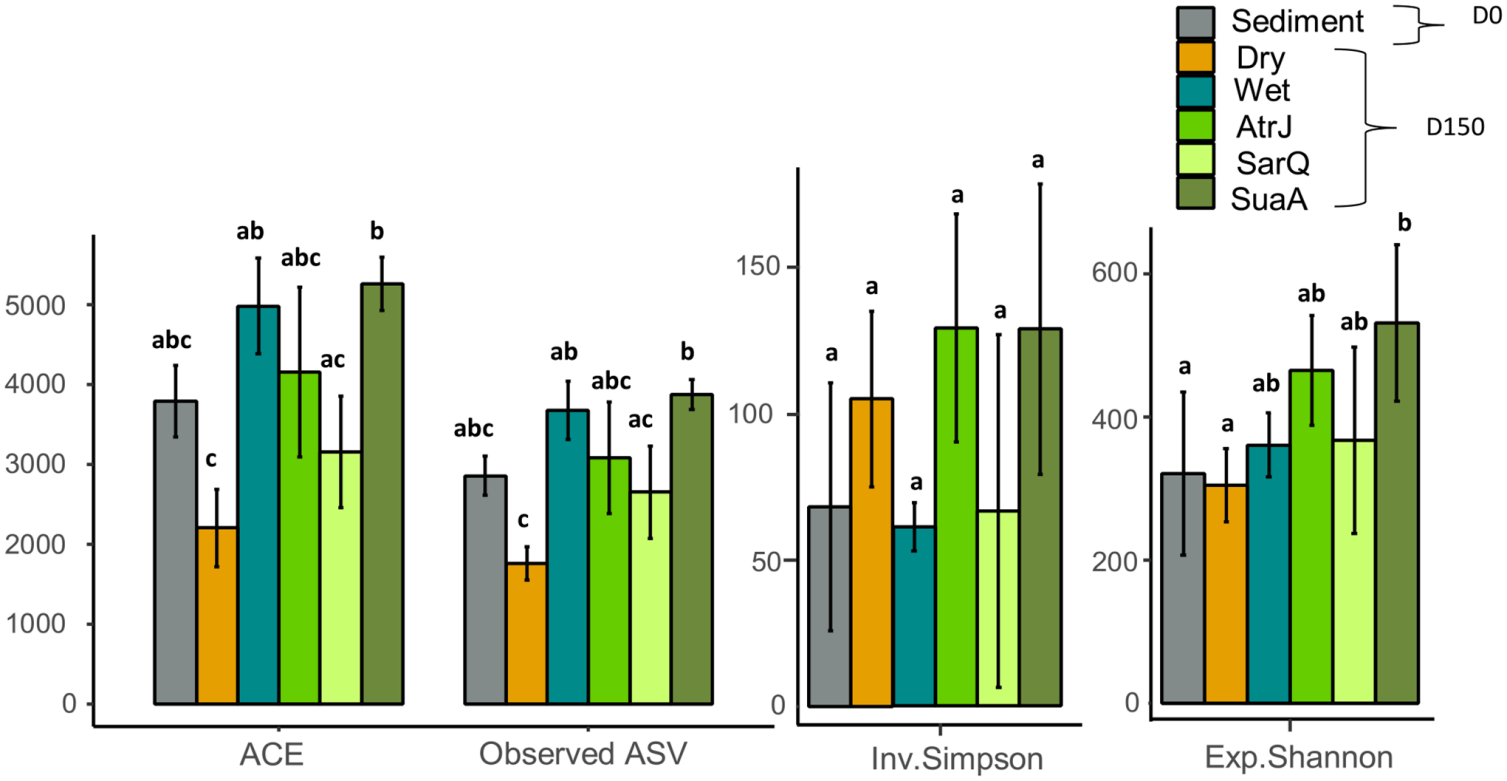
Sediment alpha diversity index at the beginning (D0) and at the end of experiment (D150) in dry, wet and halophytes (*Suaeda australis*: SuaA, *Atriplex jubata*: AtrJ, *Sarcocornia quinqueflora*: SarQ) conditions. Significant differences (p < 0.05) between conditions shown with letters at the top of the bar plot

Compared with the start of the experiment (D0), the mean values of the richness indices (ACE and ASV observed) decreased significantly in the dry sediment (Fig. 1). Inversely, sediments kept wet or with halophytes, displayed an increase of the average richness indices compared to D0. On D150, richness indices varied significantly between halophyte species and SuaA had significantly higher values than SarQ (Fig. 1). The average of exponential Shannon index increased with halophytes compared to D0, and this was statistically significant for SuaA (Fig. 1). However, for dry sediments, the exponential Shannon index remained similar to D0. The inverse Simpson index varied between the experimental conditions, but the standard deviation was too large to highlight statistical differences. The average of inverse Simpson index had increased in SuaA and AtrJ compared to D0 and also in dry sediment. Whereas, for SarQ and wet sediment, the average of inverse Simpson index was similar to D0.

### Community succession

#### Hierarchical clustering: dendrogram of sediment microbiota

**Fig. 2:**
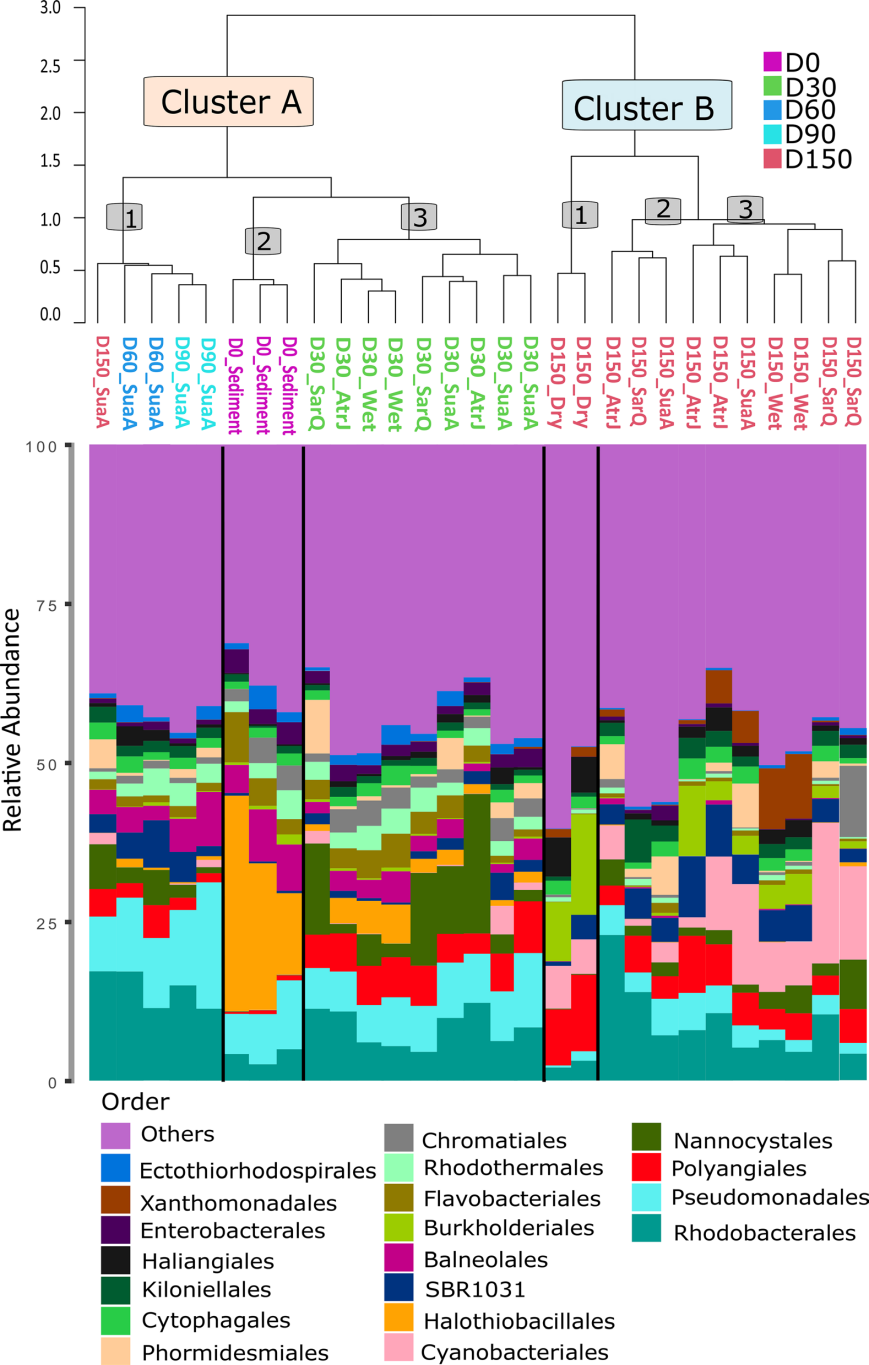
Hierarchical clustering dendrogram of sediment microbiotas following the day of experiment (D0, D30, D60, D90 and D150) and experimental conditions dry, wet, halophytes (*Suaeda australis*: SuaA, *Atriplex jubata*: AtrJ, *Sarcocornia quinqueflora*: SarQ*)*

The agglomerative Hierarchical clustering of sediment microbiota displayed two main clusters: cluster A gathered samples collected from D0 to D90, and cluster B encompassed only D150 samples (Fig. 2). According to the dendrogram, the active microbiota inhabiting the wet soil and the soil with halophytes was different between D30 and D150 (Fig. 2).

Cluster A was divided into three sub-clusters (A1 to A3). Cluster A1 gathered the active microbiota of the sediment with *Suaeda australis* collected on D60, D90 and one sample collected on D150; and was mostly made by members affiliated to the *Rhodobacterales* and *Pseudomonadales* (Fig. 2). The Cluster A2 encompassed the microbiota of sediment collected on D0 and was mostly made by members affiliated to the *Halothiobacillales*. Cluster A3, was composed by all the sediment microbiota collected on D30. This sub-clustering indicated a change of sediment microbiota compared to the beginning of experiment at D0 and D30.

Inside the cluster B, the active microbiota was separated in three sub-clusters (B1 to B3) distinguishing the microbial communities inhabiting the dry sediment (Cluster B1) from those living in wet and in the soil with halophytes (Cluster B2 and B3 respectively) (Fig. 2). Cluster B1 had greater proportion of *Burkholderiales* and *Polyangiales* whereas cluster B2 and B3 had higher proportion of *Cyanobacteriales* and *NB1_j*, a group gathering uncultured related sequences retrieved in marine microbial community [38].

### Sediment microbiota dissimilarities between dry, wet and halophytes conditions

#### Dry, wet and halophytes specific microbiota

**Fig. 3:**
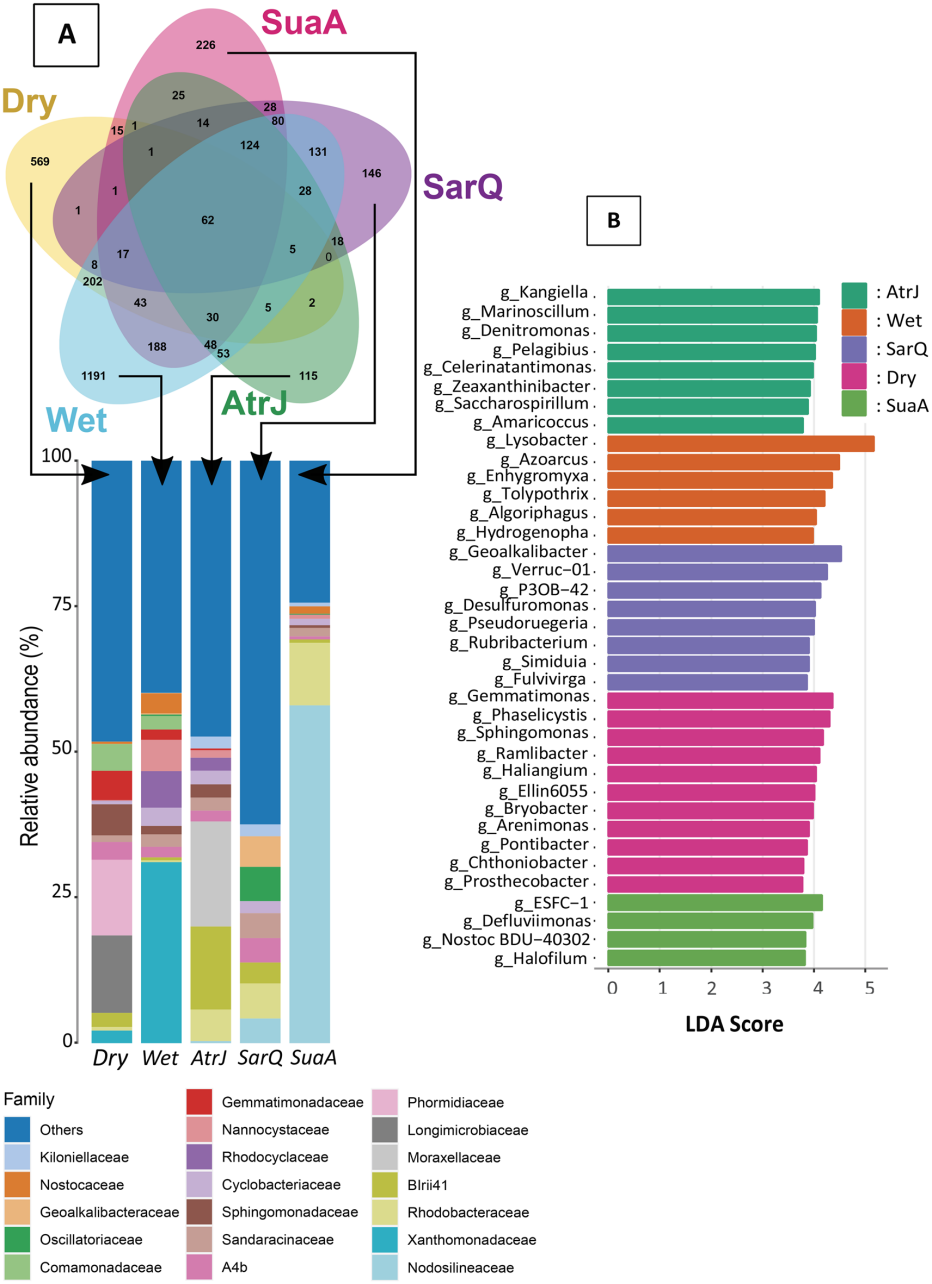
** A**: Venn diagram of shared and specific ASV from dry, wet, SuaA (*Suaeda australis*), SarQ (*Sarcocornia quinqueflora*) and AtrJ (*Atriplex jubata*) sediments. Stacked bar plot represents the relative abundance of family specific ASV. **B**: LDA score of significant sediment biomarkers at the genus level, found in dry, wet and halophyte conditions (SarQ, SuaA, AtrJ),

The Venn diagram showed that at the end of the experiment 62 ASVs were common between the microbiota of the dry, wet, AtrJ, SarQ and SuaA sediments (Fig. 3A). Dry sediment shared 202 ASVs with wet sediment but few ASVs with sediment colonized by halophytes (15 ASVs, 2 and 0 with SuaA, SarQ and AtrJ respectively). Whereas wet sediment shared 188 ASVs with SuaA, 131 with SarQ and 53 with AtrJ. Wet and dry sediments had more specific ASVs than the sediment colonized with halophytes (e.g., 1191 ASVs for wet sediment *versus* 115 ASVs for AtrJ) (Fig. 3A).

**Fig. 4:**
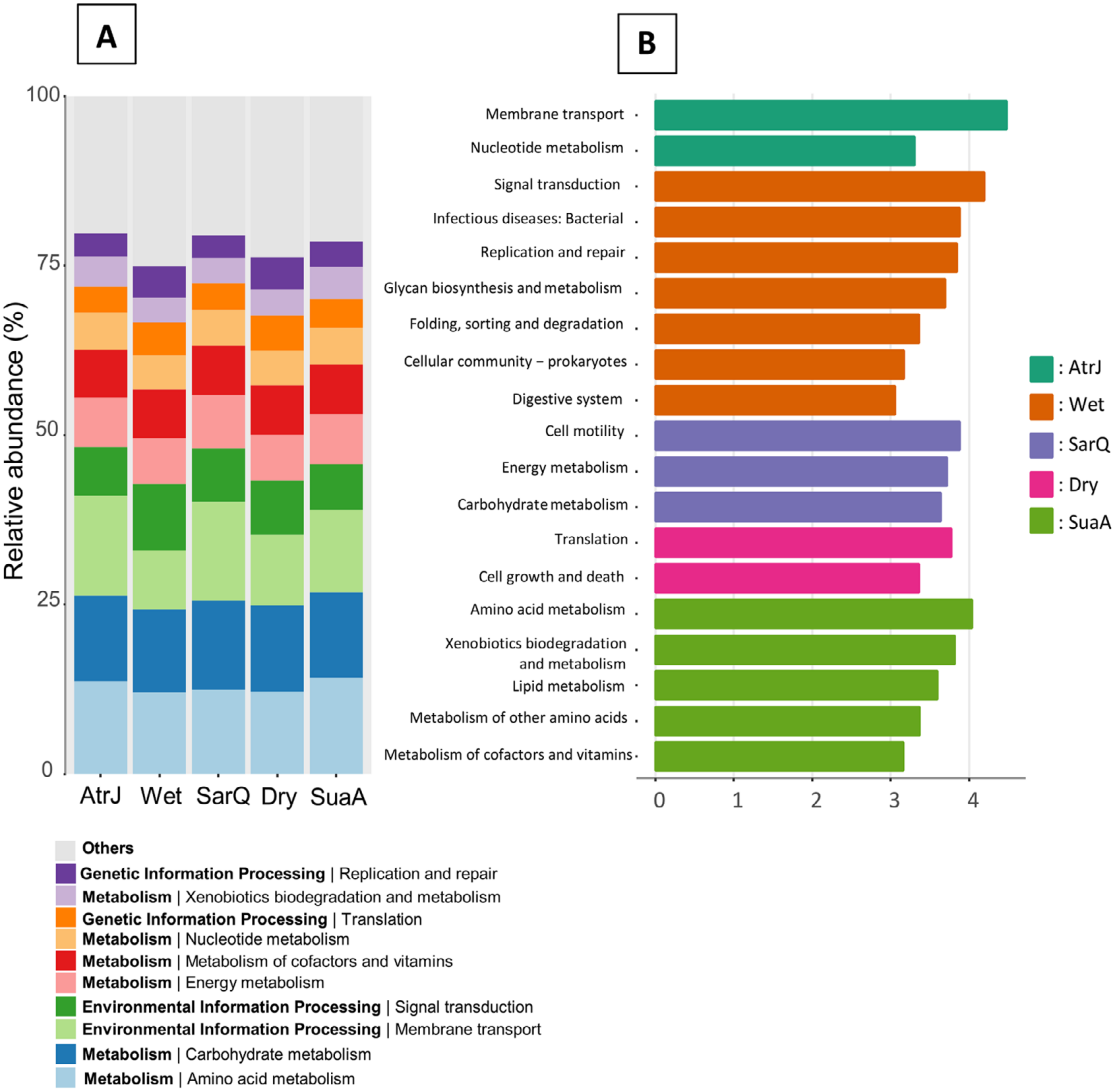
** A**; Relative abundance of functional categories found in the sediment assigned by Tax4fun tool and grouped into level 2 of KEGG Orthologues. Sediment functional categories were assigned using biomarker taxa found in Fig. 5. **B**; LDA score of significant functional categories found in the sediment following experimental conditions (dry, wet, halophyte culture)

Specific microbiota of dry sediment was mainly composed of *Cyanobacteria* (14%), *Polyangia* (13%), *Gammaproteobacteria* (10%) and *Alphaproteobacteria* (9%) classes (Supplementary Table 2 A and Additional File 1). Whereas specific microbiota of wet sediment was mainly composed of *Gammaproteobacteria* (45%) and *Polyangia* (11%). At family level, *Phormidiaceae* (13%), *Longimicrobiaceae* (13%) were specific to the dry sediment microbiota (Fig. 4A) while *Xanthomonadaceae* families were specific to wet sediment (31%).

In sediment colonized with SuaA, the specific microbiota was overpowered by the *Cyanobacteria* class (62%) (Supplementary Table 2 A, Additional File 1). *Cyanobacteria* were also present in the specific microbiota of sediment of SarQ, however in a lesser extent, with 27% of the abundance. The specific microbiota of SarQ was also compose of *Alphaproteobacteria* (14%), *Gammaproteobacteria* (13%) and *Polyangia* (8%). Contrary to the two other halophyte species, *Cyanobacteria* was not present in the specific microbiota of sediment harboring AtrJ (Supplementary Table 2 A, Additional File 1). *Gammaproteobacteria* (31%), *Alphaproteobacteria* (27%) and *Polyangia* (21%) mainly represented the specific microbiota of the AtrJ rhizosphere sediments.

At the order level, *Phormidesmiales* (17.1%) were specific to SarQ and *Moraxellaceae* to AtrJ (24%) (Supplementary Table 2B, Additional File 1). The specific microbiota of SuaA rhizosphere was dominated by *Nodosilineaceae* (58%) whereas this family was absent in AtrJ sediments and only found at about 4% in sediment with SarQ (Fig. 3A). *Rhodobacteraceae* family represented about 5 to 10% of the specific microbiota composition of the three halophytes species but was about less than 1% in dry and wet sediments.

Sediment specific microbiotas were also composed of a great proportion of “Others” families (Fig. 3A). This was particularly the case of SarQ and dry sediment for which the “Others” families represented half of relative abundance. The microbiota of the rhizosphere hosting SuaA had the less abundance of « Others » families (about 25%) (Fig. 3A).

### Sediment microbiome biomarkers

The LeFSe analysis identified 295 statistically significant biomarkers at the genus level across all samples with p < 0.05 (Kruskal-Wallis test). We have chosen to display only the genera with a minimal LDA score of 3.75 (Fig. 3B) to exhibit the more significant biomarkers.

Among specific biomarkers of wet sediment, *Lysobacter* (*Xanthomonadaceae* family), *Azoarcus* (*Rhodocyclaceae* family), *Enhygromyxa* (*Nannocystaceae* family of *Polyangia* class) and *Tolypothrix* (*Nostocaceae* family of *Cyanobacteria* class) genera had the higher LDA score (> 4) (Fig. 3B). In dry sediment, biomarkers *Phaselicystis* (*Polyangiales* order), *Gemmatimonas* (*Gemmatimonadetes* phylum), *Sphingomonas* (*Sphingomonadaceae* family) and *Ramlibacter* (*Gammaproteobacteria* class) had a LDA score > 4.

For sediment with SarQ, the most significant biomarkers were *Geoalkalibacter* (*Desulfuromonadia* class), Verruc-01 (*Puniceicoccaceae* family) and P3OB-42 (*Myxococcaceae* family) with all a LDA score > 4 (Fig. 3B). In sediment with SuaA, the main biomarkers were ESCF-1 (*Cyanobacteria*) and *Defluviimonas* (*Rhodobacteraceae*). With a lower LDA score, *Nostoc* genus was another significant biomarker from *Cyanobacteria* class (Fig. 3B). In AtrJ colonized sediment, highest LDA score were found for *Kangiella* (*Gammaproteobacteria*), *Denitromonas* (*Rhodocyclaceae* family, *Gammaproteobacteria* class), and *Marinoscillum* (*Cyclobacteriaceae* family, *Bacteroidia* class).

### Putative microbial metabolic profile associated to biomarkers at the genus level with TAX4FUN in the sediments

The relative abundance of the putative functional categories varied with the experimental conditions (Fig. 4A) and significant variation between groups were observed (Supplementary Tables 4, Additional File 1). The most abundant putative functional categories belonged to the “metabolism” module, which accounted for at least 50% in each kind of sediments (dry, wet or according to the halophyte species growing in the rhizosphere).

The LefSe analysis (Fig. 4B) shown the significantly enriched functional categories in the sediment according to the experimental conditions. Based on a minimum LDA score of 3, the highest number of significant functional categories was found in wet sediment (7), followed by SuaA (5), SarQ (3), then AtrJ (2) and dry sediments (2).

Putative functional activities in the sediment with SuaA were significantly enriched in amino acid metabolism, xenobiotic biodegradation and lipid metabolism (Fig. 4B). All functions found in sediment with SuaA belonged to the “Metabolism” category (Fig. 4B and the KO database). Putative microbial functions in the sediment with SarQ were enriched in carbohydrate and energy metabolism as well as in cell motility. Putative microbial functions in the sediment hosting AtrJ were enriched in nucleotide metabolism and membrane transport (Fig. 4B). In the wet sediment, the putative microbial functions were linked to signal transduction, replication and repair, folding, sorting and degradation. Those related functions belong to the Environmental and Genetic Information Processing categories (Following Fig. 4B and the KO database). In the wet sediment, only one function was related to Metabolism category; the glycan biosynthesis and metabolism. The putative microbial functions found in dry sediment were related to cellular processes (cell growth and death) and genetic information processing (translation). However, there was no biomarkers related to the Metabolism category (Fig. 4B).

### Putative soil function associated to taxa genus levels biomarkers with FAPROTAX

**Fig. 5:**
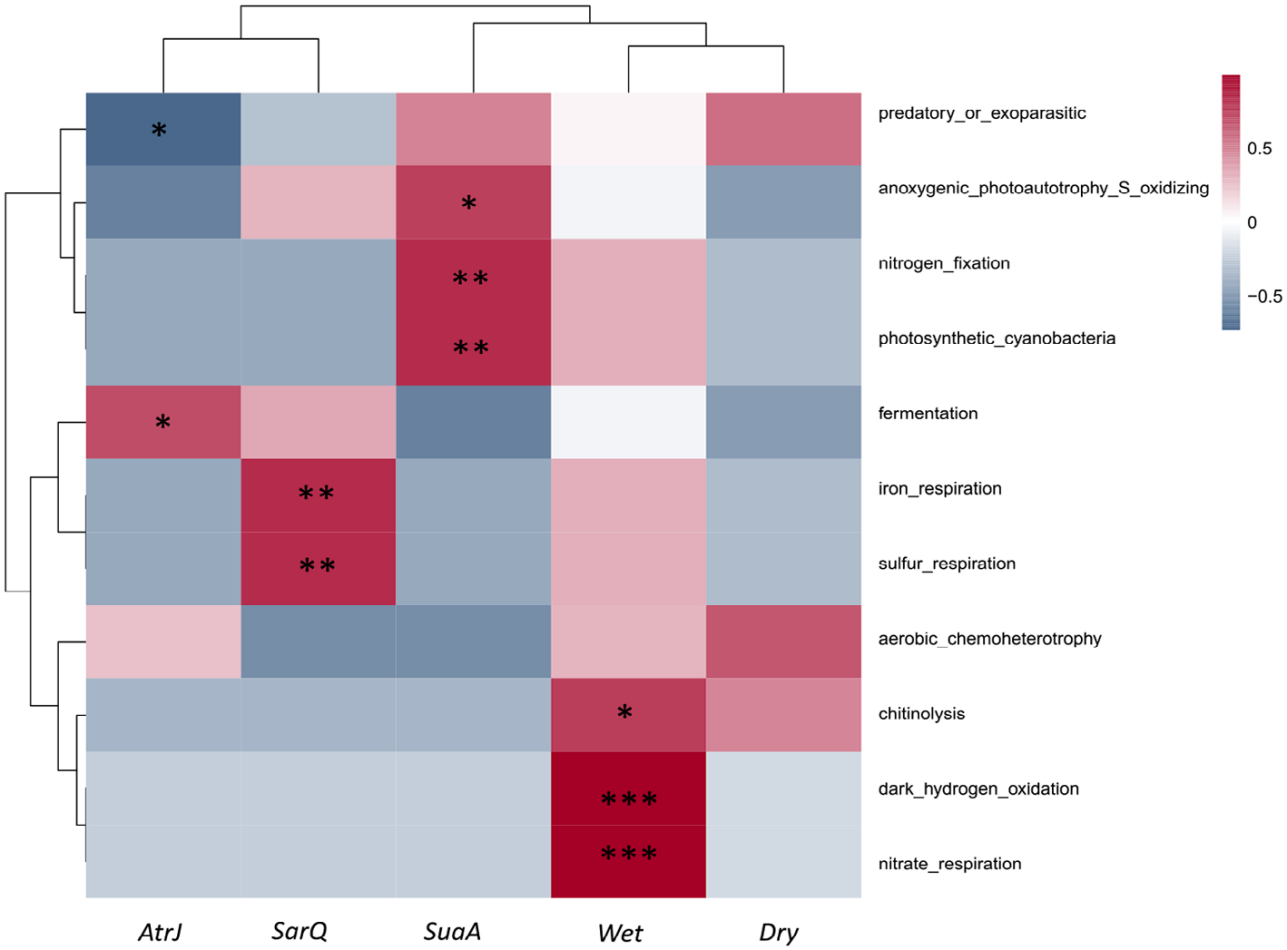
Correlogram of putative ecological functions assigned with FAPROTAX to biomarker taxa found in the rhizosphere of the halophyte species (AtrJ, SuaA, SarQ) and in wet and dry sediment. Heatmap colour gradient is link to Pearson correlation coefficient intensity with in red positive correlation and blue negative correlation. Significant correlations are indicated by an asterix (*)

Wet sediment exhibited significant correlations with nitrogen respiration, dark hydrogen oxidation and soil chitinolysis function (Fig. 5). The rhizosphere microbiota of SuaA was significantly positively correlated with nitrogen fixation, photo-autotrophy and photosynthetic *Cyanobacteria*. The microbiota of the sediment with SarQ exhibited significant positive correlation with sulfur and iron respiration functions. Whereas the microbial composition of the sediment colonized with AtrJ was positively and significantly correlated with fermentation (Fig. 5). Dry sediment showed a moderate positive correlation (Pearson correlation coefficient about 0.5) with aerobic chemoheterotrophic and chitinolytic functions, but the correlations were not significant (Fig. 5).

### Focus on suaeda australis (SuaA) microbiota succession through sampling date

#### Specific microbiota changes through time

**Fig. 6:**
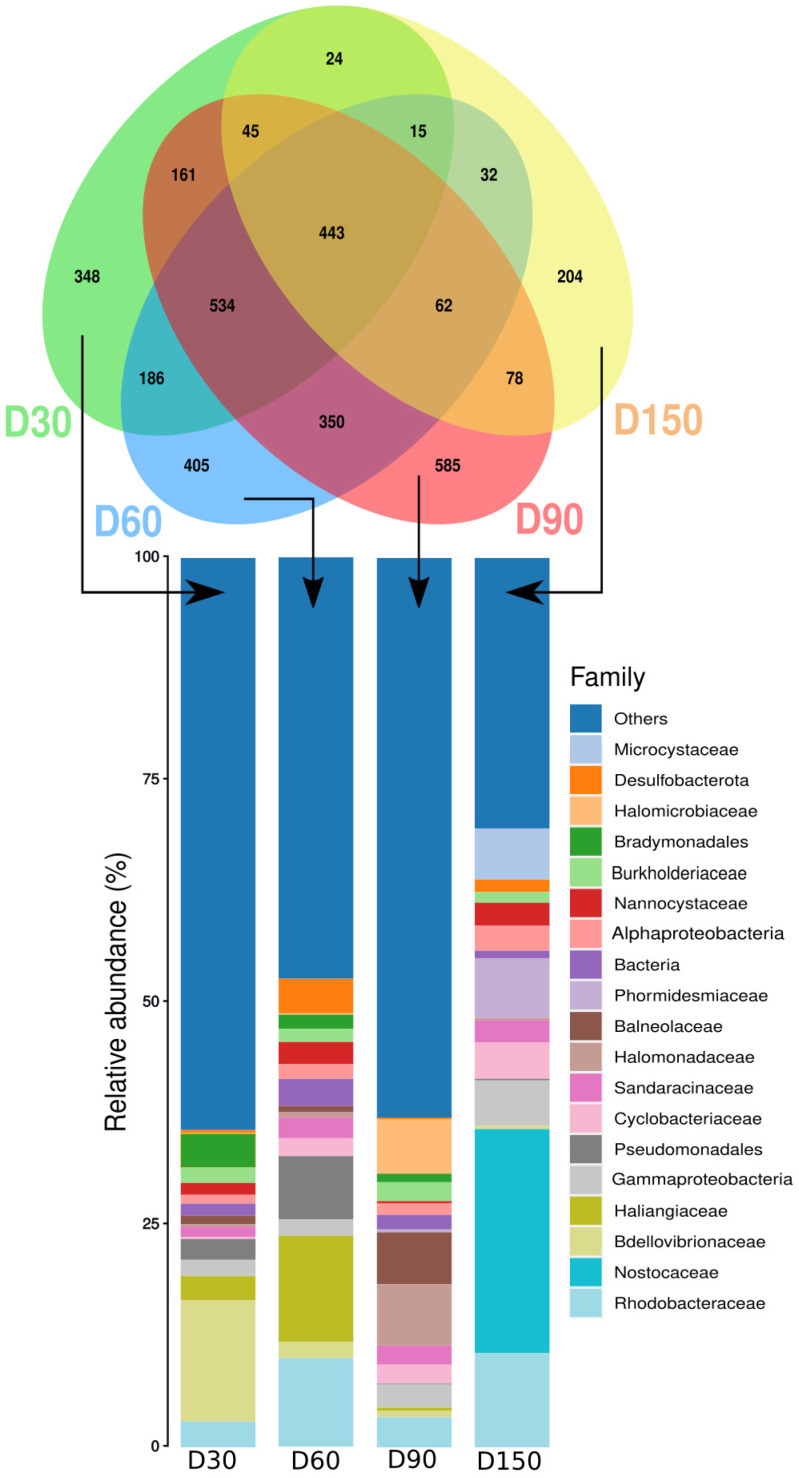
Venn diagram of shared and specific ASV from sediment with *Suaeda australis* (SuaA) through the sampling date: D30, D60, D90 and D150. Stacked bar plots represent relative abundance of specific microbiota at the family levels

Venn diagram evidenced that 443 ASVs were common between the four sampling dates (Fig. 6). When comparing samples by date, the Venn diagram showed that D60 and D90 shared the higher ASV number (350), and D150 and D30 the lower (24). D150 shared the highest ASV number with D90 (78 ASVs) compared to D60 and D30 (32 and 24 ASVs respectively), highlighting a microbial succession through the experiment and so through plant establishment (Fig. 6). That also evidenced a selection of the microbial community by the plant as only few ASVs shared between D30 and D150 (Fig. 6).

On D30, D60 and D90, *Gammaproteobacteria* represented the class with the highest relative abundance (around 20%) in the specific microbiota of SuaA (Supplementary Table 3 A, Additional File 1). On D150 *Cyanobacteria* were the main class (39%) but represented less than 0.6% in the others sampling date (Supplementary Table 3 A, Additional File 1). Following the sampling date, *Bdellovibriona*, *Polyangia* and *Halobacteria* were the other bacterial classes with the highest relative abundance. *Bdellovibriona* was approximately 16% on D30 but was less than 3% for the other sampling dates. *Polyangia* was approximately 20% on D60 and *Halobacteria* was approximately 13% at D90 but represented less than 5 and 0.2% for the other dates. At the family level, *Bdellovibrionaceae* had the highest relative abundance on D30 (14%) (Fig. 6, Supplementary Table 3B, Additional File 1); while *Haliangiaceae* (12%), *Pseudomonadales* (7%) and *Rhodobacteraceae* (10%) were the main families on D60. On D150, the specific microbiota was dominated by *Nostocaceae* family (25%). *Halomonadaceae* was specific to D90 (6%). There was also a great abundance of “Others” families in the specific microbiota as it represented more than half of relative abundance in D30 and D90 (Fig. 6).

### Biomarkers and soil putative ecological function through time

**Fig. 7:**
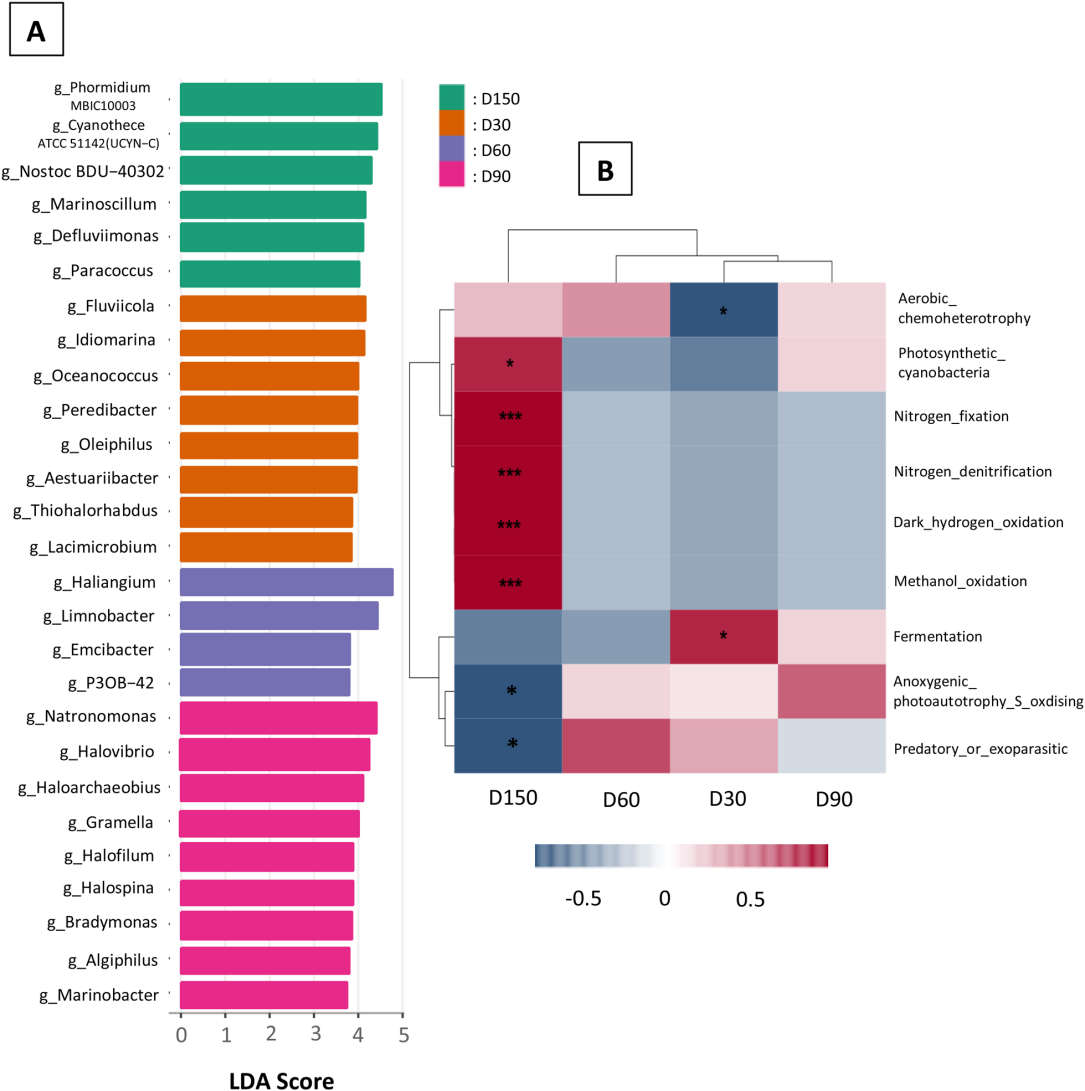
** A**; LDA score of significant sediment biomarkers found in sediment with *Suaeda australis* through the sampling time: D30, D60, D90 and D150. **B**; Correlation heatmap of putative ecological function associated with the identified biomarkers. Heatmap colour gradient is linked to Pearson correlation coefficient intensity with in red positive correlation and blue negative correlation. Significant correlations between ecological function and sampling time are indicated by an asterix (*)

The biomarker tool (LEfSe) identified a total of 200 statistically significant biomarkers at the genus level across all samples with p-value < 0.05 (test Kruskal-Wallis rank sum). Only biomarkers with a minimal LDA score of 3.75 were displayed (Fig. 7A) as they were the most significant.

On D30, *Fluviicola* and *Idiomarina* genera had the higher LDA score (> 4) (Fig. 7A). The specific microbiota on D30 was significantly positively (Pearson correlation coefficient around 1) correlated with fermentation soil function (Fig. 7B) but negatively correlated with aerobic chemoheterotrophy. *Haliangium* and *Limnobacter* had the higher LDA score on D60 (LDA > 4) and on D90, *Natromonas*, *Halovibrio* and *Haloarechaeobius* were the main biomarkers (Fig. 7A). Sediment microbiota collected on D60 and D90 were positively correlated with predatory or exoparasitic, and with anoxygenic photoautotrophy sulphur oxidizing functions respectively; however, these correlations were not significant (Fig. 7B).

On D150, three families of *Cyanobacteria* showed the highest LDA score (LDA > 4): *Phormidium, Cyanothece* and *Nostoc* (Fig. 7A). On D150, the sediment biomarkers were correlated with functions related to the nitrogen cycle (e.g., nitrogen fixation, nitrate denitrification) and methanol oxidation (Fig. 7B).

#### Evolution of the sediment content in nitrogen and organic carbon

The average NH_4_^+^ concentrations in the sediment decreased significantly at the end of the experiment. However, the final concentrations differed slightly between the modalities wet, dry and in sediment with SuaA; they were varied between 2.12 and 2.86 mg.kg^− 1^ (Table 1). After six months, NO_3_^−^ concentrations decreased from 14.65 to 12.18 mg.kg^− 1^ (approximately 17%) in dry sediment and from 14.65 to 6.11 mg.kg^− 1^ (approximately 59%) in wet sediment (Table 1). The lowest NO_3_^−^ concentration was found in the rhizosphere of SuaA with a decrease of approximately 91% compared to D0 (from 14.65 to 1.3 mg.kg^− 1^). Considering the influence of sediment humidity on NO_3_^−^ concentrations, we can deduce that halophyte culture was responsible of about 32% of sediment NO_3_^−^ concentrations decrease (halophyte 91%, wet 59%). At the end of the experiment, organic carbon has significantly increased to 10.90 mg.g^− 1^ and 9.91 mg.g^− 1^ in wet sediment and in the rhizosphere of SuaA respectively compared to the beginning of the experiment (8.61 mg.g^− 1^; Table 1). In contrast, the dry sediment concentration of organic carbon (8.60 mg.g^− 1^) was not different from the beginning of the experiment.

**Table 1:** Sediment nitrogen (NO_3_^-^ and NH_4_^+^) **±** ***SD*** and organic carbon content found at the beginning of the experiment and in dry, wet and *Suaeda australis* (SuaA) conditions at the end of the experiment (D150). Statistically significant differences (p value < 0.05) between conditions are indicated by letters

	Day of experiment	[NO_3_^−^] (mg.kg^− 1^)	[NH_4_^+^] (mg.kg^− 1^)	Organic Carbon (mg.g^− 1^)
**Sediment**	0	14.65 ± 2.52^**a**^	5.03 ± 2.78^**b**^	8.61 ± 0.83^**a**^
**Dry**	150	12.18 ± 1.07^**ab**^	2.12 ± 0.24^**ab**^	8.60 ± 0.38^**a**^
**Wet**	150	6.11 ± 1.50^**ab**^	2.04 ± 0.22^**a**^	10.90 ± 0.34^**a**^
**SuaA**	150	1.3 ± 0.21^**b**^	2.86 ± 0.31^**ab**^	9.91 ± 1.02^**a**^

## Discussion

### Influences of the sediment humidity and halophytes growth on the microbiota inhabiting the rhizosphere

In this study we choose to investigate active microbiota of the rhizosphere. In this aim, to access to the metabolically active lineages, we extracted the total RNA from our samples. Indeed, microbial diversity can be studied using either DNA or RNA, but RNA allows us to recover recent populations and living assemblages in an ecosystem [55] due to the short lifetime of RNA molecules in environment, estimated from days to weeks in soil depending on biogeochemical parameters (pH, water, temperature) [56, 57]. In addition, the high turnover rate of RNA molecules in the environment might reflect metabolically active assemblage of microbial communities at the sampling time; while microbial DNA cannot distinguish between the living and dead fraction of the microbiome. DNA therefore carries the risk to detect microorganisms that were not active or dead in the sample [55, 57]. Using alpha diversity indices, we first observed that both the growth of the halophytes and maintaining moisture favoured microbial diversity in sediment compared to sediment kept dry for the 150 days of the experiment. This is consistent with previous studies, as it is well known that humidity has a strong impact on sediment microbiota. Inversely, soil dryness can impose osmotic stress on microorganisms [5, 27, 58], and a long drought period can lead to the death of sensitive microorganisms unable to thrive in waterless conditions, resulting in a decrease in microbial diversity as highlighted by the alpha diversity indexes. Loss of microbial diversity has been demonstrated in our study as well as in others [58–60]. We also noted that the composition of microbial communities inhabiting the sediment varied significantly throughout the experiment. Indeed, based on their microbiota, there was a clear clusterization of the sediment according to the sampling period (e.g., D0, D30, D150) (Fig. 2). In addition, on D150, dry sediments are subclustered separately from wet sediment and sediment with halophytes cultivation (Fig. 2). We however did not evidence a distinct hierarchical clusterization between the wet sediment and sediments with halophyte species. Nevertheless, if considering only the specific microbiota, the Venn diagram revealed clear dissimilarities of microbiota between sediment maintaining wet, dry and with halophyte species (Fig. 3A).

The specific microbiota of *Suaeda australis* was dominated by the photoautotroph nitrogen-fixing *Cyanobacteria* bacteria with notably bacteria from *Nodosilineaceae* family (Fig. 3A). In plant-*Cyanobacteria* symbiosis, *Cyanobacteria* are reported to excrete substances (e.g. growth-promoting regulators, vitamins, amino acids) that influence plant development but also provide nitrogen sources through their abilities to fix N_2_ [61]. The prevalence of *Cyanobacteria* in the sediment with *Suaeda australis* may suggest beneficial interactions between the halophyte and the *Cyanobacteria*. This hypothesis was reinforced as *Nostoc* genus was a biomarker of this condition and is the most common *Cyanobacteria* found in plant-symbiosis [62]. The specific microbiota of *Sarcocornia quinqueflora* was also composed of *Cyanobacteria* but in lower proportions (27% compared to 62% with *Suaeda australis*) (Fig. 3A). *Cyanobacteria* was almost totally absent from *Atriplex jubata* specific microbiota that was dominated by *Alphaproteobacteria* and *Gammaproteobacteria* classes with notably *Moraxellaceae* family. This family was reported has heterotrophic bacteria found in soil and water [63]. Heterotrophic bacteria used soil organic matter, plant or animal residues as sources of energy and carbon [18]. The heterotrophic capabilities of prokaryotes may thus be relevant for degradation of accumulated organic matter in the sediment.

For the three halophytes species, we found the presence of *Rhodobacteraceae* family in their specific microbiota. This bacteria family is involved in sulphur and carbon cycles [64]. However, members of the *Rhodobacteraceae* were almost absent, with a relative abundance lower than 1%, from the specific microbiotas of the wet and dry sediments (Fig. 3A).

### Halophyte and humidity influences on sediment microbial community functions

#### Sediment metabolic profile

Plant root system provided a unique ecological niche for soil microbiota through the release of various compounds: enzyme and a wide range of molecules such as carbohydrates, amino acids or vitamins [25, 65]. The composition, diversity and abundance of roots exudates are highly plant specific; and plant species growing in a similar soil environment can recruit significantly different microbial communities [24, 66] as demonstrated previously through sediment microbial communities compositions of others halophytes species [67]. Based on KEGG orthologs, the microbial metabolic profiles found in sediment with *Suaeda australis* and *Sarcocornia quinqueflora*, were putatively linked to microbial metabolism functions related to amino acids, vitamins, carbohydrates and energy (Fig. 4B). Since plants can typically release these compounds, our results suggested that the rhizosphere microbiota of these halophytes could potentially use these substrates excreted by the plant roots. The cell motility function was also found in the sediment with *Sarcocornia quinqueflora* suggesting that microorganisms may move toward the plant root exudates. This might be important in our study as motility toward roots exudates represent the first step in rhizosphere colonization. The putative xenobiotics biodegradation metabolism found in sediment with *Suaeda australis* was previously reported as significantly enriched function in the rhizosphere of the wild blueberry and soybean [68, 69]. The enrichment of xenobiotic biodegradation metabolism in soil rhizosphere could be attributed to the release of plant-derived complex molecules [69]. Thus, the metabolic functional profiles of sediments colonized by *Suaeda australis* and *Sarcocornia quinqueflora* evidenced either a putative plant-microbe interactions or a plant attraction to select specific microbial guilds or microorganisms as shown in the first part of this discussion with the Venn diagram (Fig. 3A). In the case of sediment colonized by *Atriplex jubata*, microbial metabolic profile was putatively composed by functions related to nucletotide metabolism and membrane transport function (Fig. 4B). The putative metabolic profiles of the wet and dry sediments were mainly characterized by cellular, genetic and environmental information processing (such as replication and repair, folding, sorting and degradation) that may be vital for the proliferation and growth of microorganisms (Fig. 4B). However, contrary to the rhizospheres of *Suaeda australis* and *Sarocornia quinqueflora*, there was no evidence of functions related to metabolism except the “glycan biosynthesis and metabolisms” only for the wet sediment. Thus, the higher number of significant functions related to microbial metabolism in sediments with *Suaeda australis* and *Sarcocornia quinqueflora* may be attributed to root exudates released from plant, providing carbon and energy for microbial community activities. In the context of sediment bioremediation, this may promote organic matter decomposition activities by the microbial communities.

#### Putative sediment functional profile linked to significant biomarkers

Considering the putative functional profile of sediment based on FAPROTAX annotation, we evidenced that sediment with *Suaeda australis* was significantly linked to nitrogen fixation function (Fig. 5). This might explain the significant occurrence of phototrophic *Cyanobacteria* in sediment hosting this halophyte, including the enrichment of the *Nostoc* genus as a biomarker. Anoxygenic phototrophy sulphur oxidation was another important putative function found in the sediment with *Suaeda australis* (Fig. 5). Anoxygenic phototrophy sulphur oxidation is known to be performed by phototrophic bacteria that grow under anaerobic conditions [70]. These bacteria differ from oxygenic phototrophic bacteria by using sulphide, hydrogen or similar electron donors as the reducing power for photosynthesis rather than oxygen [70]. This function in the rhizosphere of *Suaeda australis* may be attributed to the evidence of biomarker *Halofilum*, purple sulphur bacteria, belonging to the *Ectothiorhodospiraceae* family [71]. In the context of sediment bioremediation, the hydrogen sulphide (H_2_S) oxidation by *Ectothiorhodospiraceae* family is of great interest because this compound is one of the biggest threats to aquaculture production due to its extreme toxicity to aquatic species such as shrimp [72]. Hydrogen sulphide is frequently produced in pond sediment during the sulphate reduction process using accumulated organic matter as electron donor in anaerobic condition. Thus, occurrence of the biomarker *Halofilum* in sediment with *Suaeda australis* might be relevant for sediment bioremediation. In addition, mass culture of purple and also green sulphur bacteria families as probiotics are considered as a solution to bioremediate H_2_S and maintain a favourable environment in aquaculture ponds [73–75]. Functions of the sulphur cycling had also been underlined in the sediment hosting *Sarcocornia quinqueflora*. The cultivation of this halophyte was also positively correlated with microbial sulphur and iron respiration. Sulphur respiration involves the reduction of sulphur using H_2_ or an organic substrate as electron donors [76]. This function in sediment with *Sarcocornia quinqueflora* can be explained by biomarkers enrichment related to the genera *Geoalkalibacter* and *Desulfuromonas* both belonging to the family *Desulfuromonaceae. Geoalkalibacter* and *Desulfuromonas* genera are known to reduce sulphate, and to use sulphur and metals (iron and manganese) as electron acceptors to oxidize organic compounds [60]. Sulphate-reducing microorganisms also play a relevant function as they may account for more than 50% of the organic carbon mineralization in marine sediments [77, 78]. Thus, sulphate reducers may play significant roles in both sulphur and carbon cycles during sediment bioremediation. However, as mentioned before drawback of the sulphate reducers is their production of toxic hydrogen sulphide. Although sulphur oxidation was not a significant function in sediment with *Sarcocornia quinqueflora.* The sulphate-reducers are anaerobic microorganisms that are widely spread in anoxic habitat [78]. In our experiment, the pots were sometimes waterlogged due to the low permeability of the clay sediment, which may have promoted the creation of anoxic niches in the sediment. It might also be possible that the shallow root system of *Sarcocornia quinqueflora* [16] did not enhance oxygen penetration into the sediment, and may have maintained anoxic conditions. However, the presence of sulphate-reducers such as members of the genus *Desulfobacterales* in the sediment with *Sarcocornia quinqueflora* might be due to a direct recruitment by the plant, as this genus has been previously reported being abundant in the endophytic community of another *Sarcocornia* genus growing under aquaponics conditions [13].

Contrary to the two other halophyte species, functions related to sulphur cycle were not evidenced in sediment with *Atriplex jubata*. The putative functional profile of the microbiota in the sediment with *Atriplex jubata* was significantly related to fermentation process where organic compounds are rather used as terminal electron acceptors than oxygen. The presence of this function can be explained by the biomarker *Celerinatantimonas* genus, able to use a wide variety of carbohydrates and to perform glucose fermentation. This genus also has the ability to fix N_2_ and forms apparent associations with the roots of salt marsh grasses (*Spartina alterniflora* and *Juncus roemerianus*) [79]. Biomarkers in *Atriplex jubata*, sediment are particularly marked by many chemoheterotrophic bacteria such as members of the *Kangellia*, *Marinoscillium*, *Amariccocus* and *Saccharospirillum* genera which obtained their energy through oxidation of organic compounds [18, 80]. This is the case of (i) the biomarker belonging to the genus *Kangellia* (*Gammaproteobacteria*) reported to use lignocellulose or lignocellulose-derived compounds [81], (ii) the biomarker related to the genus *Marinoscillium* (*Cytophagales*) found in decaying plant material able to degrade bio-macromolecules [82]; or (iii) the *Amariccocus* (*Rhodobacteraceae*) biomarker, a chemoheterotrophic taxon able to use a wide variety of carbohydrates and organic acids as a substrate. The significant presence of chemoheterotroph lineages in sediment colonized by *Atriplex jubata* might be related to break down organic waste (e.g., uneaten feed, faeces and dead matter) accumulated in the sediment. In addition, heterotroph lineages were also largely reported as active microbiota in shrimp pond sediment during the rearing period due to the great quantity of accumulated organic matter that was a nutrient source for bacteria [83–86].

The putative function in the wet sediment was related to chitinolysis activity, probably attributed to the detected biomarker *Lysobacter* genus (*Xanthomonadaceae* family) (Figs. 3B and 5) which has enzymatic and lytic abilities. Indeed, this genus is able to lyse several organisms such as cyanobacteria, fungi, nematodes and is also involved in the biodegradation of complex compounds as chitin [82, 87]. The detection of the chitinolytic functions was interested in our sediment as chitin is the main component of shrimp exoskeletons, which can accumulate in pond sediments after shrimp molt or dying. Indeed, in New Caledonia, when reared in earthen ponds, *Penaeus stylirostris* molt every day to every 12 days [28]. The detected putative nitrate respiration function in wet sediment can be linked to the biomarkers from the genus *Algoriphagus* (*Cytophagales* order) and the genus *Hydrogenophaga* (C*omamonadaceae* family). Members of *Algoriphagus* genus are known to be able to perform nitrate reduction (NO_3_^−^ to N_2_) [48] while taxa belonging to the genus *Hydrogenophaga* are involved in nitrate reduction through anaerobic DNRA (dissimilatory reduction of nitrate to ammonium) [88]. The nitrate reduction process in an interesting function detected here, as in New-Caledonia, shrimps are reared in semi-intensive farming; where feed pellet are distributed daily and represents the major nitrogen source in the pond. During the semi-intensive shrimp production, the shrimps assimilate solely a minor part of the pellet (about 46.7% in semi-intensive farming) [4], leading to a large accumulation of nitrogen in the sediment from uneaten feed pellet but also from faeces and dead phytoplankton. Then, denitrification process is considering as a loss of nitrogen in the environment occurrence of this function may thereby reduce nitrogen levels in wet sediment [89].

Considering the dry sediment, there is no significant putative microbial function evidenced. Its most significant biomarker was the *Gemmatimonas* genus (*Gemmatimonadetes* class) reported to be adapted to moisture-limited conditions [90]. Among the other biomarkers we evidenced the *Phaselicystis* genus that belongs to the *Polyangia* class. *Polyangia* class belongs to the *Myxococotta* phylum are well-known as micropredators able to lyse bacteria and eukaryotic organisms as well as to degrade complex macromolecules [91]. *Myxococotta* have also the particularity to form both fruiting bodies induced by nutrients deficiencies and myxospores resistant to dryness [92, 93]. Thus, dry sediment biomarkers were composed of bacteria with tolerance mechanisms to thrive under prolonged drought but no evidenced of significant taxa involved in carbon, nitrogen nor sulphur biogeochemical cycles. In accordance with Boyd and Pippopinyo (1994) [2], our results showed that at some point, drying the ponds can be detrimental to microbial activities and counterproductive to enhancing microbial decomposition of accumulated organic matter. Pond bottom should rather be dried until moisture concentrations are within the optimum range to maintain bacterial activities in the sediments. This suggestion is also reinforced by the lower values of alpha diversity index found in dry sediment compare to the others treatments.

### Focus on suaeda australis microbiota changes through time and comparison of sediment chemistry with wet and dry conditions

#### Sediment microbiota succession

The second part of this study focused on the microbial communities inhabiting the rhizosphere of *Suaeda australis* and the change of their functions through the experiment. This focus was made, as in our previous study we have demonstrated that this deep-rooted specie was efficient to perform nitrogen assimilation using compounds-derived from shrimp farming effluent [16], and because *Suaeda australis* has a greatest potential to recolonize pond bottoms during long dry periods or abandoned pond (personal observation). In addition, investigating the rhizosphere microbiota of *Suaeda australis* and its metabolic activities, was also of great interest as in New-Caledonia, this species could bring another source of economical input for the farmers. Indeed, cultivation of this halophyte during the drying period could bioremediate the pond and the leaves could also after harvest be used in shrimp feed formulation and/or in human food seasoning, as it is already done in several countries.

Then, looking at the microbiota changes in sediment harbouring *Suaeda australis*, we evidenced its dynamic through the experiment (Figs. 6 and 7A and B). This microbial shift therefore resulted in different ecological function. Thus, on D30 the microbial community was significantly correlated to fermentation function that may attributed to chemoheterotroph biomarkers *Fluviicola* (*Cryomorphaceae* family), *Idiomarina* (*Idiomarinaceae* family) and *Aestuariibacter* (*Alteromonadaceae* family) genera [94]. On D90 the microbial community was correlated to S-oxidation functions explained by the presence of the two biomarkers affiliated to the genera *Halofilum* (*Ectothiorhodospiraceae* family) and *Thiohalorhabdus* (*Chromatiales* order), bacteria capable of sulphide oxidation. On D150, microbial community was significantly correlated to nitrogen fixation and nitrate denitrification. Nitrogen fixation was related to photo-autotrophs *Cyanobacteria (Phormidium*, *Cyanothece* and *Nostoc* genera) and the nitrate denitrification to genera *Paraccocus* and *Defluviimonas* reported to reduce nitrate to N_2_ [64]. The success of sediment bioremediation is not the result of a single microbial species but must involve a microbial consortium that combined various guilds with various metabolic functions [74]. Thus, the changes of the microbial activities over time showed the implication of diverse microbial guilds that may (1) reduce the accumulated organic matter in the sediment with chemoheterotrophic bacteria activities, (2) reduce hydrogen sulphur content through sulphur oxidation and (3) reduce nitrogen level through denitrification process.

### Sediment chemistry

Regarding sediment chemistry at the end of the experimentation, the greatest nitrate reduction in sediment was observed with *Suaeda australis* compared to wet and dry sediments. In the sediment with *Suaeda australis*, the NO_3_^−^ concentration was reduced by 91% compared to the beginning of the experiment (14.56 to 1.3 mg.kg^− 1^). We could have attributed this significant NO_3_^−^ reduction solely to a plant nutrition effect; however, this hypothesis is disproven as sediments kept wet and without plant culture show a non-negligible reduction in NO_3_^−^ concentration by about 59% (14.56 to 6.11 mg.kg^− 1^). These results showed that microbial communities also have a significant role in reducing the nitrate concentration in sediment. This is coherent with the presence of taxa involved in denitrification functions in the wet sediment (*Algoriphagus* genus) and in the sediment with *Suaeda australis* (*Paraccocus* and *Defluviimonas genus*). Thus, the reduction of NO_3_^−^ in sediment by *Suaeda australis* seemed to be the result of both the plant nutrition and its rhizosphere microbiota through denitrification process. In dry sediment, the NO_3_^−^ concentration had slightly decreased about 17% compare to the beginning of the experiment. Again, this result may reflect the negative impact of sediment drying on the microbial activities compared to wet sediment or with halophyte culture.

Reducing nitrogen level in sediment through plant-microbiota associations such as *Suaeda australis* and its rhizosphere microbiota may be a relevant way of shrimp pond sediment bioremediation. Indeed, high levels of nitrogen accumulated in pond bottom are mostly due to uneaten feed, shrimp molt and faeces during the rearing [4]. In addition, accumulation of nitrogenous compounds ammonia and nitrite are toxics to the shrimps [95].

The significant reduction of NO_3_^−^ and its low residual concentrations (1.3 mg.kg^− 1^) in sediment at the end of the experiment, may explain the high abundance of active *Cyanobacteria*. This could be linked in particular to the specific microbiota inhabiting the rhizosphere with *Suaeda australis* and to the evidenced of *Cyanobacteria* as biomarkers. The *Cyanobacteria*, are oxygenic photoautotrophs with low nutritional requirement, able to fix atmospheric N_2_, conferring them a competitive advantage to colonize nutrient-poor environment [62], and then to be the main lineages at D150 in these sediments. However, *Cyanobacteria* occurrence in the sediment contributes towards in situ primary production by providing nitrogen sources and increasing the availability of natural food resources, which is another important process in aquaculture bioremediation [74]. However, it is also important to not totally removed the nitrogen from the sediment, as it is essential for development of phytoplankton involved in the primary production of the shrimp pond [1]. Shrimp pond bioremediation needs the appropriate combination of plant-microbiota association as well as sufficient sediment humidity as suggested by Boyd and Pippopinyo (1994) [2].

## Conclusion and perspectives

To conclude, we evidenced that each halophyte condition has favour both specific microbiota and putative microbial metabolism functions. This can be explained by the occurrence of plant-microorganisms’ interactions that evidenced microbial metabolisms linked to plant roots exudates compounds. In sediment kept wet, with Tax4fun we found less functions related to the category of microbial metabolisms (Fig. 6B), while in dry sediment microbial functions were only related to cellular processes and genetic information and the alpha diversity was the lowest.

In the rhizosphere of *Suaeda australis* we found functions related to denitrification and also sulphide oxidation, whereas *Sarcocornia quinqueflora* rhizosphere was related to sulphate reduction and *Atriplex jubata* to fermentation. Thus, microbial communities related to the rhizosphere of the 3 halophytes species are differently related to nitrogen, carbon and sulphur biogeochemical cycles. We can then underline that *Ectothiorhodospiraceae* family was involved in sulphide oxidation, *Geoalkalibacter* and *Desulfuromonas* genera to sulphur reduction, *Paracoccus* and *Defluviimonas* genera to denitrification, and *Celerinatantimonas* to fermentation. All these highlighted functions are relevant to the bioremediation of hydrogen sulphide, nitrogen and organic matter accumulated in shrimp ponds.

In wet sediment, microbial communities have decreased nitrogen level through denitrification functions but in sediment with *Suaeda australis* the nitrogen reduction was more important. Thus, efficiency of halophyte bioremediation is a result of both rhizosphere communities and plant nutrition. In sediment kept wet, the functions of chitinolysis and denitrification highlighted were relevant for chitin degradation and nitrogen reduction in sediment. Whereas, for dry sediment, we found a loss of microbial diversity and no microbial function link to metabolism or sediment biogeochemical cycle (using both Tax4fun and FAPROTAX). Thus, the excessive drying period of shrimp pond sediment practise by shrimp farmers may not be reliable to maintain sediment microbial decomposition activities. Therefore, the culture of halophytes in sediments seemed to be more efficient for bioremediation than dry and wet sediment, by promoting microbial activities. To go further in the analyses, it would be interesting to increase the scale and scope of this study by testing halophyte culture directly inside shrimp earthen ponds coupled to sediment geochemical and microbial metatranscriptomic analysis.

## Electronic supplementary material

Below is the link to the electronic supplementary material.

Supplementary Material 1

## Data Availability

The raw sequencing reads for this study are deposited on NCBI SRA repository available in: https://www.ncbi.nlm.nih.gov/sra/PRJNA925577.
