# Supplementary material for: Dynamic of active microbial diversity in rhizosphere sediments of halophytes used for bioremediation of earthen shrimp ponds
Source: Environ Microbiome. 2023 Jul 12;18:58. doi: 10.1186/s40793-023-00512-x (PMC10339602; doi:10.1186/s40793-023-00512-x)
Supplement: Supplementary file 1 — Supplementary Material 1 [file 40793_2023_512_MOESM1_ESM.docx]

**Additional Files 1**

**Tab.1:** Mean percentage of the main bacterial classes found in sediment at the beginning of the experiment and in sediment maintained dry. wet and with halophyte (AtrJ. SarQ. SuaA) following by the sampling day (DO. D30. D60. D90. D150).

| ***Class*** | **D30**  **AtrJ** | **D150 AtrJ** | **D30 SarQ** | **D150 SarQ** | **D30 SuaA** | **D60 SuaA** | **D90 SuaA** | **D150 SuaA** | **D30**  **Wet** | **D150**  **Wet** | **D150**  **Dry** | **DO Sediment** |
| --- | --- | --- | --- | --- | --- | --- | --- | --- | --- | --- | --- | --- |
| ***Acidobacteriae*** | 0.5 | 1.2 | 0.3 | 1 | 0.5 | 0.2 | 0.3 | 0.6 | 0.2 | 1.1 | 2.5 | 0.3 |
| ***Actinobacteria*** | 0.7 | 0.6 | 0.5 | 0.3 | 0.7 | 0.9 | 1.5 | 1.3 | 0.5 | 0.5 | 1 | 0.8 |
| ***Alphaproteobacteria*** | 21.6 | 27.9 | 18.3 | 25.9 | 19.1 | 25.9 | 23.7 | 24.1 | 13.1 | 15.5 | 10.4 | 9.2 |
| ***Anaerolineae*** | 2.7 | 7.5 | 2.1 | 4.3 | 3.7 | 6.2 | 3.6 | 4.3 | 0.9 | 6.4 | 3.2 | 1 |
| ***Bacteria*** | 1.1 | 1.2 | 0.9 | 0.8 | 1 | 1.3 | 0.9 | 0.6 | 1.3 | 0.6 | 0.1 | 0.9 |
| ***Bacteroidia*** | 5.2 | 3.7 | 6.2 | 2.6 | 4.5 | 4.2 | 3.7 | 5.3 | 9.1 | 3.9 | 4.8 | 8.7 |
| ***BD2-11 terrestrial group*** | 7 | 2.7 | 7.9 | 2.9 | 4.9 | 4.3 | 4.3 | 2.1 | 12.5 | 2.5 | 0.9 | 5 |
| ***Bdellovibrionia*** | 0.7 | 0.5 | 0.8 | 0.6 | 1.2 | 0.9 | 0.4 | 0.8 | 0.8 | 0.4 | 0.2 | 0.5 |
| ***Cyanobacteriia*** | 0.4 | 9.1 | 5.6 | 15.1 | 5.3 | 0.3 | 2.3 | 13.3 | 0.6 | 11.7 | 6.3 | 0.1 |
| ***Desulfuromonadia*** | 1.5 | 0.4 | 1.3 | 2.4 | 1.3 | 2.1 | 5.7 | 1.1 | 2.5 | 0.8 | 0.6 | 5.1 |
| ***Gammaproteobacteria*** | 23.1 | 17.6 | 19.8 | 14.6 | 26.4 | 25.1 | 28.5 | 16.3 | 28 | 23.5 | 16.8 | 46.7 |
| ***Longimicrobia*** | 0 | 0 | 0 | 0.2 | 0 | 0 | 0 | 2.1 | 0 | 0.1 | 6.3 | 0 |
| ***Myxococcia*** | 0.4 | 0.2 | 0.6 | 0.6 | 2.1 | 0.5 | 0.1 | 0.4 | 0.6 | 0.7 | 3.3 | 0.1 |
| ***NB1-j*** | 1.4 | 3 | 0.6 | 4.8 | 1.8 | 2.2 | 2.2 | 4.3 | 0.3 | 7.1 | 0.1 | 0.7 |
| ***Phycisphaerae*** | 0.2 | 0.9 | 0.2 | 0.7 | 0.2 | 0.3 | 0.3 | 0.6 | 0.4 | 1 | 3.5 | 0.2 |
| ***Planctomycetes*** | 0.5 | 0.7 | 0.3 | 0.5 | 0.4 | 0.4 | 0.8 | 0.8 | 0.5 | 0.6 | 1 | 0.4 |
| ***Polyangia*** | 17.5 | 12.1 | 21 | 10.3 | 13.1 | 10.5 | 3.8 | 9.3 | 10.5 | 10.3 | 21 | 1 |
| ***Rhodothermia*** | 4.8 | 1.2 | 5.4 | 0.7 | 4.7 | 6.3 | 10.2 | 2.2 | 7.8 | 0.9 | 0.7 | 9.5 |
| ***Verrucomicrobiae*** | 0.7 | 1.3 | 0.6 | 0.9 | 1 | 1 | 0.8 | 1.6 | 0.7 | 1.9 | 4 | 0.3 |

**Tab.2 :** Mean percentage of specific bacterial classes and families obtained with the Venn diagram in dry. wet and halophytes conditions (AtrJ. SarQ. SuaA) at the end of experiment (150 days).

**A B**

| ***Class*** | ***AtrJ*** | ***Wet*** | ***SarQ*** | ***Dry*** | ***SuaA*** | ***Family*** | ***AtrJ*** | ***Wet*** | ***SarQ*** | ***Dry*** | ***SuaA*** |
| --- | --- | --- | --- | --- | --- | --- | --- | --- | --- | --- | --- |
| *Acidobacteriae* | 1.48 | 0.41 | 0 | 2.02 | 0.07 | *A4b* | 1.9 | 1.8 | 4.2 | 3.1 | 0.5 |
| *Alphaproteobacteria* | 27.78 | 4.93 | 13.59 | 9.45 | 15.09 | *BIrii41* | 14.3 | 0.5 | 3.6 | 2.4 | 0.6 |
| *Anaerolineae* | 2.25 | 3.32 | 4.72 | 3.49 | 0.75 | *Comamonadaceae* | 0 | 2.3 | 0 | 4.6 | 0 |
| *Bacteroidia* | 4.19 | 7.94 | 6.82 | 7.14 | 1.51 | *Cyclobacteriaceae* | 2.4 | 3.1 | 2.1 | 0.5 | 1.1 |
| *BD2-11 terrestrial group* | 0 | 1.21 | 1.13 | 0.09 | 0.28 | *Gemmatimonadaceae* | 0.3 | 1.8 | 0 | 5 | 0 |
| *Cyanobacteriia* | 0.35 | 3.9 | 27.24 | 13.84 | 62.41 | *Geoalkalibacteraceae* | 0 | 0.2 | 5.3 | 0 | 0 |
| *Desulfuromonadia* | 0 | 1.16 | 7.86 | 0.79 | 2.86 | *Kiloniellaceae* | 2.1 | 0.1 | 2.1 | 0 | 0.6 |
| *Gammaproteobacteria* | 31.86 | 44.83 | 13.32 | 10.06 | 8.23 | *Longimicrobiaceae* | 0 | 0 | 0 | 13.3 | 0 |
| *Gemmatimonadetes* | 0.29 | 1.78 | 0 | 4.99 | 0 | *Moraxellaceae* | 18.1 | 0 | 0 | 0 | 0 |
| *Kapabacteria* | 0.48 | 0.15 | 0 | 1.18 | 0.3 | *Nannocystaceae* | 1.3 | 5.4 | 0 | 0.1 | 0.7 |
| *Myxococcia* | 0 | 0.25 | 2.69 | 1.4 | 0.24 | *Nodosilineaceae* | 0.4 | 0 | 4.2 | 0 | 58 |
| *OM190* | 3.15 | 0.83 | 2.29 | 0 | 0.17 | *Nostocaceae* | 0 | 3.5 | 0 | 0.4 | 1.3 |
| *Phycisphaerae* | 1.18 | 1.64 | 0.37 | 3.07 | 0.24 | *Oscillatoriaceae* | 0 | 0.3 | 5.9 | 0 | 0.2 |
| *Planctomycetes* | 0 | 0.72 | 0.73 | 0.54 | 0.17 | *Phormidiaceae* | 0 | 0 | 0 | 13 | 0 |
| *Polyangia* | 21.43 | 11.15 | 8.37 | 13.41 | 3.2 | *Rhodobacteraceae* | 5.4 | 0.3 | 6 | 0.6 | 10.7 |
| *S0134 terrestrial group* | 0 | 0.43 | 0 | 4.2 | 0 | *Rhodocyclaceae* | 2.2 | 6.2 | 0 | 0 | 0 |
| *Verrucomicrobiae* | 0.29 | 3.43 | 3.6 | 4.79 | 1.33 | *Sandaracinaceae* | 2.2 | 2.2 | 4.3 | 1.2 | 1.5 |
| *Vicinamibacteria* | 0 | 0.27 | 0 | 1.76 | 0 | *Sphingomonadaceae* | 2.2 | 1.4 | 0 | 5.4 | 0.5 |
|  |  |  |  |  |  | *Xanthomonadaceae* | 0 | 31 | 0 | 2.2 | 0 |

**Tab.3**: Mean percentage of specific bacterial classes and families bacteria obtained with the Venn diagram according to the sampling days (D30, D60, D90, D150) in sediment with halophyte *Suaeda australis*.

**A**  **B**

| ***Class*** | ***D30*** | ***D60*** | ***D90*** | ***D150*** | ***Family*** | ***D30*** | ***D60*** | ***D90*** | ***D150*** |
| --- | --- | --- | --- | --- | --- | --- | --- | --- | --- |
| Actinobacteria | 0,5 | 0,5 | 2,7 | 0,8 | *Alphaproteobacteria* | 1.0 | 1.7 | 1.3 | 2.8 |
| Alphaproteobacteria | 9,2 | 17,2 | 9,4 | 20,6 | *Bacteria* | 1.4 | 3.0 | 1.6 | 0.8 |
| Anaerolineae | 4,4 | 3,8 | 3,4 | 0,7 | *Balneolaceae* | 0.9 | 0.7 | 5.8 | 0.0 |
| Bacteria | 1,4 | 3,0 | 1,6 | 0,8 | *BD2-11 terrestrial group* | 1.7 | 1.5 | 2.1 | 1.2 |
| Bacteroidia | 9,7 | 5,9 | 6,2 | 6,3 | *Bdellovibrionaceae* | 13.6 | 1.8 | 0.7 | 0.4 |
| BD2-11 terrestrial group | 1,7 | 1,5 | 2,1 | 1,2 | *Bradymonadales* | 3.9 | 1.6 | 0.9 | 0.0 |
| Bdellovibrionia | 15,6 | 2,4 | 1,6 | 0,4 | *Cyclobacteriaceae* | 0.2 | 2.0 | 2.1 | 4.1 |
| Cyanobacteriia | 0,6 | 0,0 | 0,6 | 39,0 | *Desulfobacterales* | 0.0 | 0.1 | 0.0 | 0.0 |
| Desulfobacterota | 0,4 | 3,9 | 0,2 | 1,4 | *Gammaproteobacteria* | 1.8 | 1.9 | 2.6 | 5.1 |
| Desulfuromonadia | 4,6 | 2,4 | 5,2 | 0,2 | *Haliangiaceae* | 2.7 | 11.9 | 0.4 | 0.0 |
| Gammaproteobacteria | 24,9 | 19,9 | 23,1 | 10,4 | *Halomicrobiaceae* | 0.0 | 0.2 | 6.1 | 0.0 |
| Halobacteria | 0,1 | 0,3 | 12,5 | 0,0 | *Halomonadaceae* | 0.3 | 0.6 | 6.9 | 0.2 |
| Myxococcia | 0,1 | 1,2 | 0,0 | 2,7 | *Microcystaceae* | 0.0 | 0.0 | 0.0 | 5.8 |
| Planctomycetes | 0,1 | 0,2 | 3,4 | 0,5 | *Nannocystaceae* | 1.3 | 2.5 | 0.3 | 2.6 |
| Polyangia | 5,5 | 17,7 | 5,0 | 5,9 | *Nostocaceae* | 0.0 | 0.0 | 0.0 | 25.2 |
| Rhodothermia | 1,6 | 1,9 | 6,3 | 0,0 | *Phormidesmiaceae* | 0.0 | 0.0 | 0.3 | 6.7 |
| Sumerlaeia | 1,2 | 1,3 | 0,5 | 0,2 | *Pseudomonadales* | 2.3 | 7.1 | 0.1 | 0.2 |
| Vampirivibrionia | 4,9 | 0,1 | 0,0 | 0,0 | *Rhodobacteraceae* | 2.9 | 10.0 | 3.4 | 10.6 |
| Verrucomicrobiae | 1,0 | 1,0 | 1,9 | 2,0 | *Sandaracinaceae* | 1.2 | 2.3 | 2.1 | 2.5 |

|  | AtrJ | Wet | SarQ | Dry | SuaA |
| --- | --- | --- | --- | --- | --- |
| Metabolism\|Amino acid metabolism | ab | a | a | a | b |
| Metabolism\|Carbohydrate metabolism | ab | a | b | ab | a |
| Environmental Information Processing \| Membrane transport | a | b | ac | bc | abc |
| Environmental Information Processing \| Signal transduction | ab | c | ac | ac | b |
| Metabolism \| Energy metabolism | ab | a | b | a | ab |
| Metabolism \| Metabolism of cofactors and vitamins | a | ab | b | ab | b |
| Metabolism \| Nucleotide metabolism | a | b | ab | b | ab |
| Genetic Information Processing \| Translation | a | b | a | b | ab |
| Metabolism \| Xenobiotics biodegradation and metabolism | ab | a | a | ab | b |
| Genetic Information Processing \| Replication and repair | a | b | a | b | ab |

**Tab.4**: Statistical differences in functional categories found in sediments assigned by the Tax4fun tool. Statistical differences are indicated by letters between experimental conditions (dry, wet, halophyte).
